# Presyncope Unmasking a Massive Upper Gastrointestinal (GI) Bleed One Month After Multivessel Percutaneous Coronary Intervention (PCI) on Dual Antiplatelet Therapy

**DOI:** 10.7759/cureus.97286

**Published:** 2025-11-19

**Authors:** Dalal A Obaid, Khawla Y Alawadhi, Khalid Haiba

**Affiliations:** 1 Medicine, Mohammed Bin Rashid University of Medicine and Health Sciences, Dubai, ARE; 2 Internal Medicine, Mohammed Bin Rashid University of Medicine and Health Sciences, Dubai, ARE; 3 Intensive Care Unit, Mediclinic Parkview Hospital, Dubai, ARE

**Keywords:** antiplatelet therapy, coronary stent, dual antiplatelet therapy, gastrointestinal bleeding, percutaneous coronary intervention, proton pump inhibitor, restrictive transfusion, upper gastrointestinal bleed

## Abstract

Upper gastrointestinal (GI) bleeding is a life-threatening emergency that requires timely recognition, resuscitation, and carefully tailored transfusion strategies. Dual antiplatelet therapy (DAPT), while essential after percutaneous coronary intervention (PCI), substantially increases bleeding risk and creates a major therapeutic dilemma when severe bleeding occurs soon after stent placement.

We report an 87-year-old man with hypertension, dyslipidemia, and ischemic heart disease who presented with presyncope and dizziness one month after multivessel PCI with four drug-eluting stents. He was on DAPT with aspirin and clopidogrel. Laboratory evaluation revealed severe anemia (hemoglobin (Hb) 5.3 g/dL), and he subsequently developed hematemesis and melena with hemodynamic compromise. Urgent endoscopy identified multiple large esophageal ulcers with active bleeding, which were successfully managed with adrenaline injection. Transfusion was guided by a restrictive strategy, targeting Hb 8-9 g/dL in view of his recent cardiac history. A multidisciplinary decision was made to de-escalate antiplatelet therapy to clopidogrel monotherapy while continuing high-dose proton pump inhibitor therapy. This case underscores the complexity of balancing thrombosis and bleeding risks in the immediate post-PCI period, highlighting the role of multidisciplinary coordination, restrictive transfusion thresholds, and timely endoscopic intervention.

## Introduction

Upper gastrointestinal (GI) bleeding is a medical emergency that requires prompt resuscitation and, when indicated, urgent transfusion and intervention. Many studies have been conducted in this domain to investigate the circumstances under which transfusion is necessary. Based on recent literature, the transfusion protocol for upper GI bleeding should follow a restrictive transfusion strategy to avoid over-transfusion. In hemodynamically stable patients without significant comorbidities, the goal is to transfuse when hemoglobin (Hb) is <7 g/dL. In cirrhosis and/or variceal bleeding, it is essential to maintain a target of 7-8 g/dL. Finally, in patients with established cardiovascular disease or active ischemia, it is necessary to consider a threshold of 8-9 g/dL to transfuse. These approaches and protocols align with major gastroenterology guidelines and randomized data supporting restrictive over liberal transfusion strategies, which improve patient survival and outcomes [1].

There are many common causes of upper GI bleeding, including peptic ulcer disease (often due to Helicobacter pylori or chronic nonsteroidal anti-inflammatory drug use (NSAID) use), erosive esophagitis/gastritis, varices, Mallory-Weiss tears, and, less commonly, malignancy and angiodysplasia. Urgent endoscopy is required, and high-dose proton pump inhibitor (PPI) therapy is recommended once the patient is stabilized.

However, when upper GI bleeding occurs in patients receiving dual antiplatelet therapy (DAPT) after coronary stenting, management becomes significantly more complex. Clinicians face a therapeutic dilemma, balancing the need to prevent life-threatening stent thrombosis against the risk of recurrent bleeding. While antiplatelet agents do not directly cause mucosal injury, they impair platelet aggregation and delay clot formation, thereby exacerbating bleeding when mucosal lesions or ulcerations are present [2]. Conversely, discontinuing DAPT prematurely can lead to stent thrombosis or myocardial infarction, underscoring the importance of multidisciplinary decision-making between cardiology and gastroenterology teams.

The American College of Gastroenterology - Canadian Association of Gastroenterology (ACG-CAG) guideline advises against discontinuing antiplatelet therapy in the setting of GI bleeding, emphasizing that aspirin should be resumed promptly for secondary cardiovascular prevention once endoscopic hemostasis is achieved. Although aspirin itself poses a higher risk of mucosal injury compared to other agents, discontinuation of aspirin markedly increases the risk of thrombotic events; thus, the benefits of early reintroduction outweigh the risks in most patients. Contemporary cardiology literature supports the use of short-course DAPT followed by monotherapy in selected high-bleeding-risk patients [3].

## Case presentation

This case describes an 87-year-old man who presented to the emergency department with constant dizziness while walking. He remained fully conscious throughout the episode. He was pale and tachycardic upon arrival. He denied any chest pain, dyspnea, headache, nausea, vomiting, visual change, or change in bowel habits. Past medical history included hypertension, dyslipidemia, and ischemic heart disease. One month earlier, he had undergone coronary angiography showing three-vessel disease requiring percutaneous coronary intervention (PCI), receiving four drug-eluting stents (DES), and was discharged on DAPT consisting of aspirin and clopidogrel. 

**Figure 1:**
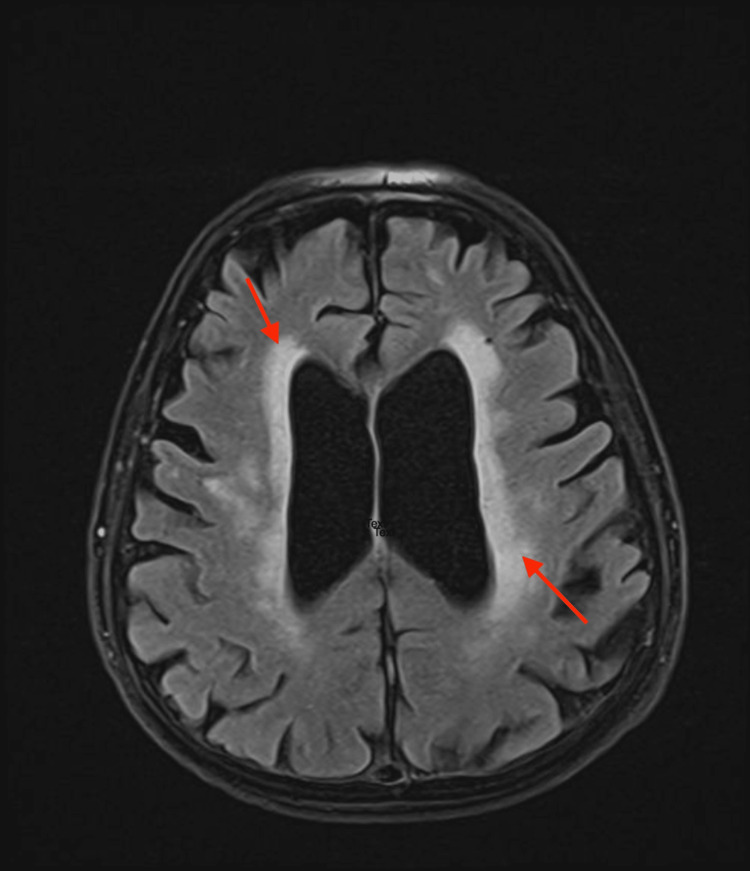
Axial FLAIR MRI of the brain showing periventricular white matter changes Axial FLAIR MRI demonstrates bilateral periventricular hyperintense lesions surrounding the lateral ventricles (arrows), consistent with chronic small vessel ischemic changes. The ventricles appear mildly enlarged with prominence of cortical sulci, suggestive of cerebral atrophy. No evidence of acute ischemia. FLAIR, fluid-attenuated inversion recovery

Upon physical examination, the patient was alert and oriented, with a Glasgow Coma Scale score of 15/15. She was hemodynamically stable with a blood pressure of 118/76 mmHg, heart rate of 112 beats per minute, respiratory rate of 18 breaths per minute, oxygen saturation of 98% on room air, and temperature of 36.8°C. Orthostatic vital measurements were unremarkable, showing no significant postural drop in blood pressure. Systemic examination, including cardiopulmonary, abdominal, and focused neurologic assessments, was otherwise unremarkable. Given her recent PCI and ongoing antithrombotic therapy, neurology was consulted to exclude an acute cerebrovascular event, as the reported “dizziness” could have represented a presyncopal episode rather than a primary neurological symptom. CT angiography of the head and neck revealed atherosclerotic changes without flow-limiting stenosis. Given the acute presentation, a brain MRI was performed for its higher sensitivity, demonstrating global cortical and subcortical atrophy with extensive white-matter hyperintensities consistent with chronic small-vessel ischemic disease, and no evidence of acute infarction (Figure 1).

Initial laboratory investigations revealed profound anemia, with a Hb level of 5.3 g/dL and a lactate concentration of 5 mmol/L. Comprehensive laboratory findings at presentation are summarized in Table 1.

**Table 1:** Laboratory values on presentation The table displays the patient’s laboratory results at presentation alongside standard reference ranges. All measurements are reported in conventional units.

Laboratory Marker	Patient Value	Reference Range
Hemoglobin	5.3 g/dL	13.0-17.5 g/dL
White Blood Cell Count	22.1×10³/µL	4.0-11.0×10³/µL
Neutrophil Count	81% (17.9×10³/µL)	43.5-73.5% (1.7-7.6×10³/µL)
Platelet Count	218×10³/µL	150-450×10³/µL
International Normalized Ratio/Prothrombin Time	1.33/17.5 sec	0.80-1.20/11.7-15.3 sec
Activated Partial Thromboplastin Time	29.3 sec	28.6-40.0 sec
Serum Creatinine	125 µmol/L	71-115 µmol/L
Estimated Glomerular Filtration Rate	48 mL/min/1.73m²	>60 mL/min/1.73m²
Blood Urea	21 mmol/L	2.9-8.2 mmol/L
Serum Sodium	142 mmol/L	136-145 mmol/L
Serum Potassium	4.8 mmol/L	3.5-5.1 mmol/L
Serum Chloride	106 mmol/L	98-107 mmol/L
Serum Bicarbonate	23 mmol/L	22-28 mmol/L
Blood Lactate	5.0 mmol/L	0.5-2.0 mmol/L
C-Reactive Protein	16 mg/L	0-5 mg/L
High-Sensitivity Cardiac Troponin	14.5-15.3 ng/L	<14 ng/L

Shortly after, he developed hematemesis and melena with hypotension and marked pallor. An upper GI bleed protocol was activated. Antiplatelets and prophylactic enoxaparin, which had been initiated for venous thromboembolism prevention during hospitalization, were withheld. High-dose intravenous pantoprazole was initiated, and he was resuscitated with packed red blood cells. He was transferred to the intensive care unit on a 3 L/min nasal cannula for close monitoring and supportive care. Urgent oesophagogastroduodenoscopy (OGD) evacuated approximately 800 mL of blood and clots and revealed large, multiple coalescing esophageal ulcers. Adrenaline (1:100,000) was injected in and around the ulcers with successful hemostasis. The target Hb range was maintained between 8 and 9 g/dL.

On day 2, repeat OGD showed regressing and healing esophageal ulcers, a large hiatus hernia, and mild gastritis/duodenitis with no active bleeding. Hb increased with transfusion (from 6.9 to 8.8 g/dL), mean arterial pressure stabilized at 70 mmHg, and there were no recurrent bleeding episodes. Subsequent laboratory results following transfusion and stabilization are presented in Table 2.

**Table 2:** Laboratory values after stabilization Laboratory results of the patient after stabilization compared with standard reference ranges. All values are expressed in conventional units.

Laboratory Marker	Patient Value	Reference Range
Hemoglobin	8.8 g/dL	13.0-17.5 g/dL
White Blood Cell Count	15.2×10³/µL	4.0-11.0×10³/µL
Neutrophil Count	76.8% (11.7×10³/µL)	43.5-73.5% (1.7-7.6×10³/µL)
Lymphocyte Count	11.7% (1.8×10³/µL)	15.2-43.3% (1.0-3.2×10³/µL)
Platelet Count	190×10³/µL	150-450×10³/µL
International Normalized Ratio/Prothrombin Time	1.33/17.5 sec	0.80-1.20/11.7-15.3 sec
Activated Partial Thromboplastin Time	29.3 sec	28.6-40.0 sec
Serum Creatinine	122 µmol/L	71-115 µmol/L
Estimated Glomerular Filtration Rate	49 mL/min/1.73m²	>60 mL/min/1.73m²
Blood Urea	13.6 mmol/L	2.9-8.2 mmol/L
Serum Sodium	142 mmol/L	136-145 mmol/L
Serum Potassium	4.8 mmol/L	3.5-5.1 mmol/L
Serum Chloride	107 mmol/L	98-107 mmol/L
Serum Bicarbonate	23.6 mmol/L	22-28 mmol/L
C-Reactive Protein	18 mg/L	0-5 mg/L
High-Sensitivity Cardiac Troponin	20 ng/L	<14 ng/L

The cardiology team advised de-escalating DAPT to single antiplatelet therapy (SAPT). The patient was maintained on clopidogrel 75 mg daily and discontinued aspirin. The plan also included continuing esomeprazole 40 mg daily for gastroprotection upon transitioning from intravenous to oral therapy. Serial cardiac biomarkers and electrocardiographic monitoring did not suggest an acute coronary event, and no immediate cardiac intervention was required. Neurology found no objective deficits and attributed transient dizziness to anemia and hypoperfusion.

With clinical improvement, the patient was planned for transfer from the intensive care unit to the medical ward under the gastroenterology team, with coordinated outpatient follow-up. He was continued on high-dose PPI therapy and clopidogrel monotherapy.

## Discussion

According to the American Heart Association (AHA), DAPT is indicated after PCI with the placement of a DES, such as the Ultimaster Tansei, as used previously in our patient in this context. DAPT lowers the risk of stent thrombosis and recurrent ischemic events, and typically consists of low-dose aspirin (81 mg daily) combined with a P2Y12 inhibitor (clopidogrel 75 mg daily, ticagrelor 90 mg twice daily, or prasugrel 10 mg daily). 

The recommended duration is at least six months for stable ischemic heart disease and 12 months for acute coronary syndrome (ACS). In patients with high bleeding risk, shorter courses of one to three months may be considered, particularly with newer-generation DES such as the Ultimaster Tansei. These stents feature thinner struts and biocompatible polymer coatings that promote faster endothelial healing and reduce the incidence of late stent thrombosis, thereby allowing for earlier de-escalation of DAPT without compromising long-term safety [4].

In our patient, DAPT was initiated following multivessel PCI. However, significant upper GI bleeding developed one month later, necessitating re-evaluation of the antiplatelet regimen. Based on the MASTER DAPT trial, early de-escalation from DAPT to SAPT can be justified in patients with high bleeding risk once hemostasis is secured. This approach balances the competing risks of stent thrombosis and recurrent bleeding. In line with cardiology recommendations, aspirin was discontinued, while clopidogrel was continued, along with the initiation of gastroprotective therapy [5]. Management decisions were made collaboratively through a multidisciplinary team (MDT) discussion between cardiology, gastroenterology, and critical care, during which the characteristics of the Ultimaster Tansei stent, specifically its newer-generation design supporting shorter DAPT duration, were key factors in safely transitioning the patient to SAPT. Notably, the patient’s initial presentation with dizziness was later identified as presyncope rather than a primary neurological symptom, underscoring how transient hemodynamic instability can mimic neurological complaints in patients on antithrombotic therapy.

Transfusion management was guided by the International Consensus Group recommendations (Barkun et al.), which advocate for individualized thresholds based on comorbidities and ischemic risk. In hemodynamically stable patients without significant cardiovascular disease, Hb should generally be maintained above 8 g/dL. In contrast, patients post-PCI are at a higher ischemic risk, which warrants a higher target of >9 g/dL. Guidelines support more restrictive strategies (Hb >7 g/dL) for patients with suspected variceal bleeding. Finally, more liberal thresholds (>10 g/dL) may be appropriate in ongoing active bleeding or concurrent myocardial ischemia. In our patient, transfusion decisions reflected his dual risk profile, minimizing recurrent bleeding while ensuring adequate oxygen delivery given his recent coronary stenting [6].

## Conclusions

This case highlights an elderly patient who, one month after multivessel PCI on DAPT, presented with presyncope that initially prompted a neurologic work-up to rule out stroke but rapidly declared itself as a life-threatening upper GI bleed from multiple esophageal ulcers, causing severe anemia and requiring urgent transfusion. Endoscopic hemostasis, transfusion, and high-dose PPI therapy achieved stabilization. This case highlights the importance of an MDT in achieving the best patient outcome, which in this case required safely transitioning from DAPT to single-agent therapy just one month post-PCI to balance the risks of thrombosis and bleeding appropriately.
